# Comparing Single-Item and Multi-Item Trust Scales: Insights for Assessing Trust in Project Leaders

**DOI:** 10.3390/bs13090786

**Published:** 2023-09-21

**Authors:** Marcela Souto Castro, Bouchaib Bahli, João J. Ferreira, Ronnie Figueiredo

**Affiliations:** 1 Instituto Politécnico de Setúbal, Escola Superior de Ciências Empresariais (ESCE), Campus do IPS Estefanilha, 2914-503 Setúbal, Portugal; 2 Research Center for Business Sciences (NECE), Universida de Beira Interior (UBI), 6201-001 Covilhã, Portugal; jjmf@ubi.pt (J.J.F.); ronnie.figueiredo@ipleiria.pt (R.F.); 3 Information Technology Management, Toronto Metropolitan University, 350 Victoria Street, Toronto, ON M5B 0C3, Canada; bahli@torontomu.ca; 4 Centre of Applied Research in Management and Economics (CARME), School of Technology and Management (ESTG), Polytechnic of Leiria, 2411-901 Leiria, Portugal; 5 Spinner Innovation Centre (SIC), 2840-640 Setúbal, Portugal

**Keywords:** trust, single-item, measurement, scale, multi-item, project members

## Abstract

The purpose of this research is to provide researchers and leaders with a reliable and up-to-date comparison between a single-item and a multi-item trust scale, enabling effective assessment of team members’ trust in their leaders. The aim of the study is to investigate whether a single-question scale is as reliable as a multi-item questionnaire in measuring trust. An additional goal is to provide researchers with insights and conditions for effectively using single or multiple measures to assess trust in leaders, considering factors like reliability and effectiveness. After conducting a comprehensive literature review, data were collected from 101 project members in Brazil using a survey methodology. The respondents were asked to provide feedback regarding their leaders, specifically project managers, and factor analysis was then employed to test the single-item and multi-item measures of trust. The advantages and disadvantages of each approach are discussed. The findings of our study demonstrate that both single-item and multi-item scales of trust should be utilized to gain a more comprehensive understanding of the trust construct. Single-item questionnaires can reduce survey length, improve respondent friendliness, and increase participant willingness. On the other hand, multi-item questionnaires enable researchers to analyze latent variables that contribute to an overall variable, but they cannot isolate data for each of those constructs. The results show that both measures are reliable, providing researchers and professionals with insights into the benefits and drawbacks associated with each method. Consequently, this research equips researchers and project professionals with valuable information for selecting the appropriate measurement tool.

## 1. Introduction

Trust is an important competence in leaders’ relations with team members, other peers, and customers and is crucial for engagement and, hence, organizational success. Conceptually, trust facilitates communication and engagement and encourages the generation of ideas and innovation.

Over the past three decades, especially after the publication of a seminal paper on measure development [1], the adoption of multi-item (MI) measures in academic research has surged, especially with the emergence of advanced modeling techniques such as structural equation modeling [2].

Nevertheless, some management researchers frequently highlight that empirical research in organizations usually faces the challenge of time and willingness to respond to surveys [3,4]. They argue that researchers need to make trade-offs between (1) reasonable questionnaire length, (2) value of additional information, (3) data quality, (4) research costs, (5) response rate, and (6) study results. Therefore, while academic researchers prefer an MI scale measurement for their constructs, some practitioners favor single-item (SI) measures to minimize respondent refusal and research costs [4]. Therefore, developing and validating MI measures for use in academic research makes no sense in terms of practical issues, as SI measures are preferred in practice [5].

When surveys are lengthy and non-user-friendly, a smaller proportion of the target audience is willing to respond [4,6]. There is a strong, positive correlation between drop-off rates and the number of questions in a survey [7]. Hence, there is a need for design solutions for questionnaires so researchers can collect information faster, more cheaply, and accurately. Shorter questionnaires have the potential to obtain higher-quality information from respondents [8].

For some time, there have been concerns about the validity of single-item indicators. Doubts have been raised as to whether they can accurately capture the critical factors conceptualized as part of the examined construct or whether they should be abandoned in favor of an MI scale. It is well known that among academics, SI measures are usually avoided because of their presumed low reliability, although this low reliability is not a concrete issue [5,9,10]. However, contrary to the premise that MI measures are generally superior to SI measures, the publication by Bergkvist and Rossiter [11] has challenged this tradition on both theoretical and empirical grounds and concludes that “theoretical tests and empirical findings could be unchanged if good single-item measures were substituted in place of commonly used multi-item measures.” The use of SI measures has been investigated and encouraged by several researchers who consider this kind of measure appropriate and that it can substitute MI measures in many cases. For instance, Graf et al. [12] found that 64% of articles in psychology use an SI questionnaire to measure trust. SI measures have already been confirmed as reliable and useful in fields such as health, psychology, management, and marketing research [10,13,14]. Given these opposing views, and with the objective of contributing to the accumulation of knowledge, the aim of this study is to provide researchers with some insights and conditions under which the appropriate use of single or multiple measures can be adopted.

To achieve our research goal, we compared an SI scale to an MI trust construct in IT project leaders. The project management context was selected as a convenience sample due to the authors’ ease of access to professionals in this field. Additionally, the relevance of this context stems from the nature of projects as temporary organizations in which leaders are required to establish trust rapidly. Therefore, trust plays a fundamental role in project environments while being crucial in any relationship and context. For the purpose of our study, we used the most widely cited trust construct that consists of three dimensions (16 items): ability (six items), benevolence (five items), and integrity (five items) [15,16]. To the best of our knowledge, no other study in project management research has made this comparison.

In the past decade, there have been a vast number of publications on trust as a main construct. When pre-existing measures are available, researchers often borrow other researchers’ ad hoc scales to conduct surveys. According to McEvily and Tortoriello [16], in 48 years of management literature review, only 24 previously developed and validated measures of trust have been replicated, and 11 of these replications were by the same authors who originated the measure, demonstrating that the trust construct measurement is rudimentary and highly fragmented. Furthermore, in the management literature, there is weak evidence in support of the validity of the trust construct and limited consensus on operational dimensions [16].

The contributions of this study are twofold. First, to accumulate knowledge in this area, the objective is to compare SI and MI scales of the trust construct in project leaders and to provide statistical evidence of the possibility of narrowing empirical research questionnaires on trust without the empirical analysis losing reliability.

Second, to improve practice for academics, this study provides alternatives for measuring trust with SI and/or MI measures. They will have the option of choosing the most appropriate measure of trust that fits their research objectives, target audience, and questionnaire design. This possibility also allows professionals to assess stakeholders’ level of trust, whether they are a team or any different stakeholder.

This paper is organized as follows: the next section describes the theoretical background upon which this study is built. Section 3 presents the research methodology used. Section 4 represents the data analysis and the study results, followed by a discussion and conclusion. We conclude this paper with the implications of the study’s findings on both research and practice.

## 2. Theoretical Background

The significance of trust between team members and the team leader has been extensively documented. Previous research studies indicate that trust among partners enhances team knowledge sharing [17], team commitment [17], the ability to focus on project work [18], and team and individual performance [19]. Team efficacy relies upon trust and collaboration among team members, as it can enhance their willingness to share knowledge and engage in open discussions to address issues and manage conflicts [20].

Three decades ago, it was argued that the use of an SI as a measure is contingent upon how unambiguous and concrete the construct is to the respondent [21]. Later, several researchers offered evidence of similar predictive validity of an SI predictor when compared to an MI scale [11,22,23]. It was stated that “carefully crafted single-item measures—of doubly concrete constructs—are at least as valid as multi-item measures of the same constructs, and that the use of multi-items to measure them is unnecessary” [24] (p. 618). Given recent concerns regarding “over-surveying,” decreasing response rates, in order to minimize respondent refusal and estimate the costs of data collection, [25], the temptation of using SI for its advantages of parsimony and ease of administration is appealing [26]. Graf et al. [12] found that the SI scale is as valid as the MI measure, possibly due to the high reliability of the MI scale. That is, the very high internal consistency (i.e., Cronbach’s alpha) may indicate an unnecessary duplication of content across items and thus points to redundancy rather than homogeneity [27]. Hence, the items of an MI scale may all measure the very same thing and, therefore, do not show higher predictive validity than the SI scale.

In contrast to their perceived practical advantages, SI measures are often considered unreliable and invalid. Graf et al. [12] demonstrated that MI measures consistently outperform SIs, raising questions about recent research findings. Academics typically avoid SI measures [3,4,7], and MI measures are more commonly accepted for journal publications due to their presumed higher reliability [3,28]. This is based on the Spearman-Brown prophecy—the statistical effect through which measurement error in the total scale score of an MI scale decreases as random measurement errors cancel each other out when averaged across items [5]. Simulation studies by Diamantopoulos et al. show that MI scales generally outperform SI in terms of predictive validity, except under very specific conditions [29]. Therefore, researchers must exercise caution when abandoning established MI scales in favor of SI measures, as the predictive validity of SIs can vary across constructs [30]. Kwon and Trail [31] found mixed results, with MI scales sometimes performing better, sometimes showing no difference, and sometimes being outperformed by SI measures. Therefore, the use of SI measures in empirical research should be approached cautiously and limited to special contexts.

Conversely, practitioners often prefer SI measures to minimize respondent refusal and researcher costs [3,4]. Additionally, empirical research conducted in organizations faces time constraints and respondents’ willingness to complete lengthy questionnaires. Traditions can have dual effects: (1) knowledge accumulation, building upon previous work; (2) hindrance of inquiry, potentially necessitating fresh perspectives for refining or improving knowledge [32].

It is a notorious barrier to academics and practitioners collecting enough data and obtaining a good sample. A small quantity of data gathered in research may decrease reliability and sometimes render research analysis impossible. Questionnaire length and a respondent-friendly questionnaire design have been proven to influence survey response [6,7,33]. The longer the survey, the less willing the target audience is to respond.

Adıgüzel and Wedel [8] tested two versions of the same questionnaire, a full version with 65 questions and a reduced version with 33 questions. These authors found the following: (1) a 25% decrease in the questionnaire response time, from eight to six min; (2) there were 33 skip responses for the full questionnaire and five for the reduced one, mostly occurring at the last half of the questionnaire; (3) the quality of the data obtained from the reduced questionnaire tended to be better than that from the full questionnaire; and (4) reduced questionnaires were evaluated more positively by respondents and led to a significant reduction in boredom and fatigue.

Using SI measures may imply less time for respondents to complete questionnaires and may be more flexible than MI scales [33,34], yet the benefits of SI measures go beyond time constraints and respondents’ willingness to respond. The use of SI measures has been investigated and encouraged by several authors who consider this type of measure as appropriate and capable of substituting multi-item measures in many contexts [5,13]. In a frequently cited study, [35] compared multiple- and SI questionnaires to measure job satisfaction, and they concluded that one-item measure is superior to an MI facet scale. According to these authors, MI measures may neglect some components of job satisfaction that are important to an individual employee or may include components unimportant to him. In other words, there are individual differences in what constitutes job satisfaction among different employees [34]. Since job satisfaction may vary for each individual, summing up facets that are unimportant and neglecting the ones that are considered important to measure satisfaction will lead to misleading conclusions about a job satisfaction level because global measures of job satisfaction are not equivalent to the sum of the facet satisfaction [35]. Wanous, Reichers, and Hudy [9], and Nagy [34] subsequently confirmed the convergent validity of the Scarpello and Campbell [35] study years later, reinforcing the argument of the SI measure for job satisfaction and concluding that an SI scale has proven to be more robust than facet measures.

Cheah et al. [36] support the use of SI measures in small sample research of hospitality management due to their degrees of convergent validity compared to MI tools. Although MI measures have been shown to have greater convergence validity for larger sample sizes, differences are marginal. Hence, the use of SIs to measure the criterion construct is encouraged [36].

Gilbert and Kelloway [37] suggested six SI measures of job stressors that performed almost as well as the MI measures in explaining the criterion supporting their use as SI facet measures. In Kulikowski’s [15] study on the work engagement construct, which is also a psychological construct, the reliability of the SI measure ranged from 0.6 to 0.7. Despite the lower reliability of an SI measure in comparison to MI, it can still provide useful information because, in a structural equation model, latent variables of job resources and burnout could similarly predict work engagement, whether it was quantified via SI or MI measurements [15]. SI measures of job stressfulness can identify cases of common mental disorders in working populations [38] and can also predict sick leave, depression, and exhaustion [39]. A life satisfaction SI was also confirmed as reliable in comparison with the MI measure [14].

The SI measure was also tested in marketing research. Bergkvist and Rossiter [40] compared SI and MI measures to assess attitudes toward an advertisement, a brand, and purchase intentions at an interval of two years [24]. The authors found no difference in results between the two types of measures. Drolet and Morisson [3] posit that the incremental information gained from adding items to an MI scale is extremely small in marketing research. In advertising studies, when an attitudinal construct is double concrete—with a clear singular meaning in which the object being rated is also clear and singularly identifiable—it can be measured with an SI [33].

In psychology, the measurement of social identification—the notion that individuals categorize themselves into specific social identities, resulting in the tendency to behave as a collective group as opposed to individuals—has been validated as a reliable SI measure [41]. Similarly, the measurement of psychological strain as an SI measure has been confirmed to be more time and cost-efficient than MI measures and may also have psychometric benefits [37]. For example, an SI measure of self-esteem demonstrates good psychometric quality in terms of convergent validity [42].

According to Wanous et al. [9], SI measures can be categorized into two categories: (a) self-reported facts, such as gender, age, and country, and (b) narrow or ambiguous psychological constructs related to individual expectancies, such as job satisfaction. In the COAR-SE procedure for scale development designed by Rossiter [23], constructs such as beliefs, perceptions, intentions, and satisfaction can be generalized to attitudes and do not require MIs to represent them in the measure. Providing no comprehensive empirical or theoretical justification, the COAR-SE procedure discourages Likert scales on an SI measure based on the argument that there is no “psychological zero.” Against this unbased statement, Alexandrov [13] conducted a comprehensive study on Likert scale characteristics using an SI, clarifying the behavior of Likert measures, providing practical recommendations, and highlighting that a Likert scale should be positively worded with a fairly high level of intensity.

On the other hand, MI measures are apparently considered more reliable because they enable the computation of correlations between items [4]. The higher the alpha coefficient, the higher the correlation, indicating that all items represent the construct attribute if one-dimensionality is also confirmed. However, “if the attribute of the construct is concrete, alpha reliability is not a relevant criterion for evaluating the measure” [11]. Therefore, using an MI scale unnecessarily may increase costs and time, increase the effort of gathering data and analysis, and disturb respondents without providing benefits. The use of MIs to measure concrete constructs is not justifiable to capture more information [11]. Thus, several authors offered solid arguments for SI measures [7,11,23], not to mention that MI scales may diminish the quality of responses and provide very little additional information compared to a single- or, at most, two-item scale [3,4].

Trust is a fundamental element that underpins successful leadership and has emerged as a prominent area of inquiry in the realm of leadership research. Trust in leadership reflects the belief employees have in their leaders’ competence, reliability, integrity, and benevolence. Empirical studies have consistently demonstrated the positive impact of trust on various organizational outcomes, such as employee satisfaction, commitment, and performance. For instance, there is a significant influence of trust in facilitating effective communication, collaboration, and knowledge sharing within teams, thereby promoting innovation and organizational adaptability [43]. This finding is aligned with the fact that trust has a critical role in leadership in building a harmonious work environment and enhancing team cohesion [44].

In addition to the aforementioned studies, further evidence supports the positive relationship between transformational leadership behaviors and followers’ affective trust in work and task performance. Altunoğlu, Şahin, and Babacan [45] conducted a study that provided evidence of this relationship, highlighting the significant impact of transformational leadership on followers’ trust and the subsequent outcomes. Similarly, M. Nazmul Islam, Fumitaka Furuoka, and Aida Idris [46] explored the relationship between transformational leadership, trust in leadership, and employee championing behavior during organizational change. Their findings revealed a positive association between transformational leadership and trust, emphasizing the importance of trust in facilitating employee-championing behavior in times of change.

These studies underscore the crucial role of transformational leadership in cultivating trust among followers and its subsequent influence on employee outcomes. Transformational leaders who exhibit inspirational qualities and motivate their subordinates can foster a sense of trust, enabling employees to perform at their best and contribute to the organization’s success. Trust in leadership acts as a crucial catalyst that influences employee attitudes and behaviors, ultimately impacting organizational performance and effectiveness. Regarding the usage of trust measures in academic studies, we can highlight some recent quantitative researchers: [47] used a two-item trust measure; [48,49] used a five-item trust measure; [50] a seven-item trust measure; and [51] a twenty-item trust measure. These examples illustrate, first, the significance of the trust construct in academic and practical fields. Second, studies using different measures are examples of the diversity and the lack of consensus regarding the trust construct.

Based on the conflicting viewpoints presented above, the aim of this study is to contribute to the accumulation of knowledge on SI vs. MI measures of trust by examining trust in leaders. By comparing an SI trust measure to MI scales, this study provides researchers and practitioners with some insights on the appropriate selection of a scale that better suits their purpose in terms of reliability and effectiveness.

## 3. Materials and Methods

**Figure 1:**
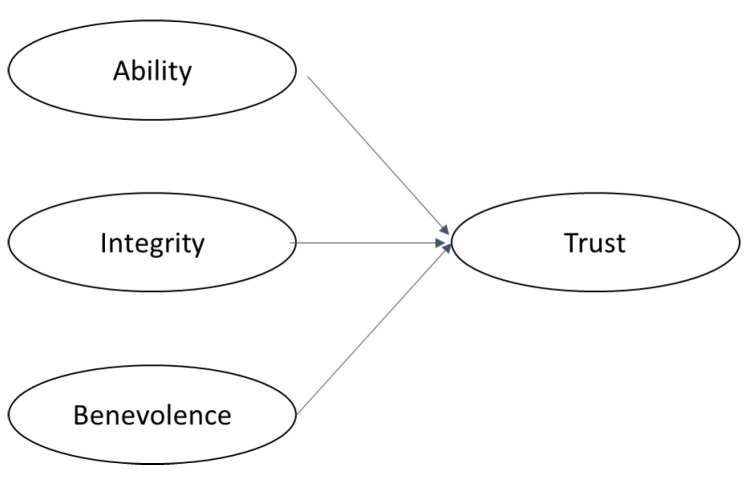
Trust dimensions.

The “two-step” approach was used to validate the trust construct formed by the three dimensions (Figure 1): ability, benevolence, and integrity (see Appendix A). Thus, the first-order latent variables were computed from a factor analysis, and these formed the second-order construct. For the confidence construct formed directly by the four items (TR1, TR2, TR3, and TR4, also described in Appendix A), the factor analysis was applied only once because it was a first-order construct. For the single-question trust construct (In general, I trust the project manager), the process of scale validation was necessary since it is not a latent variable.

The Kaiser–Meyer–Olkin (KMO) measure of sample adequacy was used to determine the proportion of variance in the data that might be common across all variables. To evaluate the quality of the indicators created by the factorial analysis to represent each question, the reliability and convergent validity of each one were verified. To measure reliability, Cronbach’s alpha (C.A.) and composite reliability (C.R.) were used. Spearman’s correlation coefficient and correlation test were used to measure the correlation between confidence indicators. The software used in the analysis was R (version 3.4.0).

### 3.1. Data Collection and Sample Characteristics

Data was gathered through a survey of project team members in Brazil from May to August 2022. A questionnaire was sent randomly to 500 project team members associated with the Project Management Institute (PMI) in Brazil. The questionnaire was designed to measure how project team members trust their leaders (project managers). The complete questionnaire can be found in Appendix A. A total of 101 project team members from different teams with different leaders returned the completed questionnaire. The sample characteristics are listed in Appendix B.

Concerning data usage consent, the introduction of the questionnaire requested consent for research purposes. Every publication resulting from this survey dealt with aggregated interpretations of the respondents’ answers so that the answers to this questionnaire will be kept strictly confidential and will never be disclosed in isolation.

### 3.2. Measurements

McEvily and Tortoriello [16] present a systematic review of trust measurement research. They identified 129 different techniques to measure trust, of which only 22 have been replicated more than once. The most replicated trust measure is the leader measure questionnaire in academic studies that was developed by Mayer et al. [52], who proposed trust as being comprised of three factors: ability, benevolence, and integrity. Originally, this instrument was used to measure how subordinates trust their leaders in organizations. In our study, we substituted the word leaders for IT project managers.

This research compares a questionnaire using three dimensions of the latent variable trust (benevolence, integrity, and ability) to the direct trust measure questionnaire developed by these same authors. Ability—six items were used to assess the team’s perception of the leader’s ability. Benevolence—five items were used to assess the team’s perception of leader benevolence. Integrity—six items were used to assess the team’s perception of leader integrity. Trust—four items were used to measure trust in a leader. We then compare the MIs with an SI measure of trust added to the original questionnaire by the authors. The items used in the questionnaire can be found in Appendix A. All constructs in the survey were measured using MI scales with a five-point Likert rating system.

## 4. Data Analysis and Results

### 4.1. Validation and Creation of the First- and Second-Order Confidence Indicators

Factorial analysis was used to validate the second-order constructs (ability, benevolence, and integrity); convergent validity, reliability, and dimensionality of the constructs were observed. Convergent validity ensures that the indicators of a construct are correlated enough to measure the latent concept. Reliability reveals the consistency of measurements in measuring the concept they intend to measure, and dimensionality indicates how many concepts the construct is measuring.

A criterion proposed by [53] was used to test the convergent validity of the constructs. C.A. and C.R. indicators were used to measure the reliability of the constructs. To verify the dimensionality of the constructs, the Kaiser criterion was used. Before validating constructs, the factorial loads, commonalities, and weights of each item of the construct were analyzed. At this step, we tried to identify whether all the items are correlated with the latent construct, that is, the concept they are forming. In other words, factorial loads indicate the degree to which an item can explain the concept or latent construct. According to Hair et al. [54], items with factorial loads under 0.50 should be eliminated since they do not contribute significantly to the formation of the latent variable.

Table 1 indicates the weights, factorial loads, and commonalities of the constructs. Accordingly, it should be emphasized that only the item IN4_inv had a factorial load below 0.50 and so will be excluded from the integrity construct.

Table 2 demonstrates the results of the convergent validity, reliability, and dimensionality analyses of the constructs. It can be concluded that (1) all constructs reached the required levels of reliability since the reliability indexes C.A. and C.R. were higher than 0.60; (2) according to Kaiser’s criterion, all the constructs were one-dimensional; (3) AVE values were higher than 0.40 in all constructs, proving the convergent validation of all of them; and (4) in all constructs, the factor analysis adjustment was adequate since all KMOs were greater than or equal to 0.50.

#### 4.1.1. Second-Order Trust Construct

Table 3 represents the weights, factorial loads, and the commonalities of the construct indicators of the second-order trust construct. Obviously, it can be emphasized that all three indicators had factorial loads above 0.50, as recommended.

As results of the convergent validity, reliability, and dimensionality analyses, it can be concluded that (1) the required levels of reliability were reached since the reliability indexes C.A. (0.88) and C.R. (0.87) were higher than 0.60; (2) according to Kaiser’s criterion, the construct was one-dimensional (dimensionality = 1); (3) the extracted variance value (0.80) was higher than 0.40, indicating convergent validation; and (4) the factorial analysis adjustment was adequate since the KMO (0.68) was greater than 0.50.

#### 4.1.2. First-Order Trust Construct

Table 4 indicates the weights, factorial loads, and commonalities of the items of the first-order trust construct. It should be highlighted that the item “TR3- Inv: I really wish I had a good way to keep an eye on a project manager” was excluded from the construct because its factorial load was below 0.50 and so contributed very little to the construction of the first-order trust construct.

As a result of convergent validity, reliability, and dimensionality analyses, it can be concluded that (1) although the required level of reliability was not reached by the C.A. index (0.46), it was reached by C.R. (0.66); (2) according to Kaiser’s criterion, the construct was one-dimensional (0.54); (3) the extracted variance value (0.49) was higher than 0.40, thus showing convergent validation; and (4) the factorial analysis adjustment was adequate since the KMO (0.54) was greater than 0.50.

### 4.2. Correlation of Indicators

To ascertain the correlation between the indicators, Spearman’s coefficient and correlation tests were used. It should be noted in Table 5 that all three confidence indicators were positively and significantly correlated, and the second-order trust indicator x single-variable trust indicator had the strongest correlation (0.84).

## 5. Discussion and Conclusions

Empirical research usually faces the barrier of time available for respondents to fill out questionnaires, especially in a business environment. There is a strong positive correlation between drop-off rates and the number of questions in a survey [7]. The longer the survey, the less willing the target audience is to respond [6], which may sometimes decrease reliability [8] and disable research analysis. This “conventional tradition” has led some researchers to use SI scales. The use of SI measures has been investigated and encouraged by several researchers who consider this kind of measure appropriate and that it can substitute MI measures in many instances.

To contribute to this debate, the aim of this study is to provide researchers with insights and conditions under which the appropriate use of single or multiple measures in terms of reliability and effectiveness could measure trust in leaders.

To achieve our research goal, we compared SI to MI trust constructs in IT project managers. For MI measures, we applied the most widely cited trust construct, which consists of three dimensions (17 items): ability (six items), benevolence (five items), and integrity (five items) [15,16]. To the best of our knowledge, there has been no previous study in project management that attempted this comparison.

The three dimensions of the second-order trust construct—ability, benevolence, and integrity—were tested as sufficient to measure trust. The required levels of reliability were reached since the reliability indexes C.A. and C.R. were higher than 0.60; the construct is one-dimensional. According to Kaiser’s criterion, the extracted variance value was higher than 0.40, showing convergent validation, and the factorial analysis adjustment was adequate since the KMO was greater than 0.50. Thus, these three dimensions proved to be reliable in measuring trust.

The confidence of the first-order trust construct, formed directly by four items, was also shown as a reliable measure of trust after deleting the item (CO_3_). However, the comparison between first-order and second-order trust constructs, considering extracted variance, C.A., C.R. and the Kaiser–Meyer–Olkin measure of sample adequacy, leads to the conclusion that the second-order trust construct is more consistent to use in surveys.

The three dimensions of the second-order measure of trust were shown to be more consistent in measuring trust. However, comparing the correlation between the three indicators (single variable, first-order, and second-order), the second-order and single variable were those that presented the highest correlation, supporting the hypothesis of similar results between them. Therefore, when it comes to lengthy questionnaires or space restrictions, the single variable measure can be used as a consistent alternative.

In addition, previous studies also validated SI measures for a variety of psychological constructs, namely organizational justice, life satisfaction, social identification, fatigue, self-efficacy [15], job satisfaction [35], hospitality management [36], stress [15,37,38], and attitude toward advertising or brands [40].

Researchers need to have the option of choosing the best measure that fits their research objectives, target audience, and questionnaire design. Short questionnaires offer the potential to obtain higher-quality information from respondents [8]. These research findings can enable the shortening of questionnaires and facilitate the application of surveys. Therefore, in alternative to the three dimensions of trust (twelve items), an SI measure is recommended to be added to the questionnaire.

The contribution of this study is to provide both researchers and practitioners with the possibility of narrowing empirical research questionnaires without losing the reliability of empirical analysis. Researchers will be able to select the most appropriate measure of trust in the leader that fits their research objectives, target audience, and questionnaire design. For professionals, this study provides alternatives or a combination of measuring trust using both an SI and an MI measure. As trust among partners and leaders may enhance knowledge sharing, team commitment, and performance, this measure can be used by organizations to test, understand, and reinforce relationships between team members.

Nevertheless, some limitations must be considered. Although using an SI instrument may adequately serve the purpose of obtaining comprehensive data for a multidimensional variable, it restricts researchers’ ability to consider each dimension individually. A researcher who wishes to examine latent constructs that contribute to an omnibus variable cannot isolate data for each of those constructs.

Another limitation is the fact that this survey focused on the project environment and measured how project team members trust their project manager. The measure testing in this specific context is important because different contexts may result in varying salience of constructs, which may impact measure structures [55]. By definition, a project is a temporary system undertaken to create a product, service, or result. A project team is usually multifunctional and composed of members with different skills and knowledge backgrounds, and a project manager should be able to extract the best performance from them. A project manager has the challenge of integrating the knowledge and skills of his team members in a short period of time to achieve project goals and deliver the expected results. These temporary and sometimes rapidly formed relationships may interfere with trust. It is suggested that trust measures be tested on other target audiences as well.

## Figures and Tables

**Table 1:** Factorial load, commonality, and weight of the second-order trust construct.

Item	Initial			Final		
	FL ^1^	Com. ^2^	Weight	FL	Com.	Weight
AB1	0.91	0.83	0.20	0.91	0.83	0.20
AB2	0.89	0.79	0.20	0.89	0.79	0.20
AB4	0.88	0.78	0.20	0.88	0.78	0.20
AB6	0.85	0.72	0.19	0.85	0.72	0.19
AB3	0.83	0.70	0.19	0.83	0.70	0.19
AB5	0.82	0.68	0.18	0.82	0.68	0.18
BE4	0.91	0.82	0.28	0.91	0.82	0.28
BE1	0.87	0.76	0.26	0.87	0.76	0.26
BE2	0.86	0.74	0.26	0.86	0.74	0.26
BE3	0.75	0.56	0.23	0.75	0.56	0.23
BE5	0.63	0.40	0.19	0.63	0.40	0.19
IN3	0.92	0.85	0.25	0.93	0.86	0.25
IN1	0.87	0.75	0.23	0.87	0.76	0.24
IN2	0.87	0.75	0.23	0.86	0.75	0.24
IN5	0.81	0.66	0.22	0.82	0.68	0.23
IN6	0.76	0.57	0.20	0.77	0.59	0.21
IN4-inv	0.36	0.13	0.10	-	-	-

^1^ Factorial load; ^2^ Commonality.

**Table 2:** Construct quality measures.

Construct	Items	EV ^1^	C.A. ^2^	C.R. ^3^	K.M.O. ^4^	Dim ^5^
Ability	6	0.75	0.93	0.91	0.92	1
Benevolence	5	0.66	0.86	0.86	0.79	1
Integrity	5	0.73	0.90	0.89	0.84	1

^1^ Extracted variance; ^2^ Cronbach’s alpha; ^3^ Composite reliability; ^4^ Kaiser–Meyer–Olkin measure of sample adequacy; ^5^ Dimensionality.

**Table 3:** Factorial Load, commonality, and weight.

Second-Order Construct	First-Order Construct	F.L. ^1^	Com. ^2^	Weight
Trust	Ability	0.94	0.89	0.39
	Benevolence	0.87	0.76	0.36
	Integrity	0.87	0.76	0.36

^1^ Factorial load; ^2^ Commonality.

**Table 4:** Factorial load, commonality, and weight of the first-order trust construct.

Item	Initial			Final		
	F.L. ^1^	Com. ^2^	Weight	F.L. ^1^	Com. ^2^	Weight
TR4	0.81	0.66	0.54	0.81	0.66	0.55
TR2	0.70	0.49	0.47	0.71	0.51	0.49
TR1-Inv	0.52	0.27	0.35	0.55	0.31	0.38
TR3-Inv	−0.26	0.07	0.17	-	-	-

^1^ Factorial load; ^2^ Commonality.

**Table 5:** Descriptive measures of the confidence indicator.

Variables	r	*p*-Value
Second-Order Trust Indicator × First-Order Trust Indicator	0.55	0.000
Second-Order Trust Indicator × Single-Variable Trust Indicator	0.84	0.000
First-Order Trust Indicator × Single-Variable Trust Indicator	0.65	0.000

r = correlation coefficient.

## Data Availability

The data presented in this study are available by email request: marcela.castro@esce.ips.pt.

## Appendix A. Questionnaire

**2nd-Order Construct**	**1st-Order Construct**	**Items**	**Description**
Trust	Ability	AB1	The project manager is very capable of performing his/her job.
		AB2	The project manager is known to be successful at the things he/she tries to do.
		AB3	The project manager has a lot of knowledge about the work that needs to be done.
		AB4	I feel very confident about the project manager’s skills.
		AB5	The project manager has specialized capabilities that can increase our performance.
		AB6	The project manager is well qualified.
	Benevolence	BE1	The project manager is very concerned about my welfare.
		BE2	My needs and desires are very important to the project manager.
		BE3	The project manager would not knowingly do anything to hurt me.
		BE4	The project manager really looks out for what is important to me.
		BE5	The project manager will go out of his/her way to help me.
	Integrity	IN1	The project manager has a strong sense of justice.
		IN2	I never have to wonder whether the project manager will stick to his/her word.
		IN3	The project manager tries hard to be fair in dealings with others.
		IN4_Inv	The project manager’s actions and behaviors are not very consistent. *
		IN5	I like the project manager’s values.
		IN6	Sound principles seem to guide the project manager’s behavior.
-	Trust	TR1_Inv	If I had my way, I wouldn’t let the project manager have any influence over issues that are important to me. *
		TR2	I would be willing to let the project manager have complete control over my future in this company.
		TR3_Inv	I really wish I had a good way to keep an eye on the project manager. *
		TR4	I would be comfortable giving the project manager a task or problem which was critical to me, even if I could not monitor their actions.
-	Single Question	SQ	In general, I trust the project manager.
		* Inverted Questions.	

## Appendix B. Sample Characteristic

**Sample’s Characteristic**		**N ^1^**	**%**
Time working with projects	Less than 1 year	13	13%
	Between 1 and 2 years	38	38%
	Between 2 and 5 years	28	28%
	Between 5 and 10 years	14	14%
	More than 10 years	8	8%
Sector of activity	Agribusiness	2	2%
	Trade/service	48	48%
	Industry	32	32%
	Mixed	19	19%
Organization	Third sector	6	6%
	State-owned	1	1%
	Private	83	82%
	Public	11	11%
Business area	Consulting	14	14%
	Engineering and construction	20	20%
	Innovation and technology	25	25%
	Internal projects in the organization	16	16%
	Others	26	26%
Projects per year	Less than 10	33	33%
	Between 10 and 50	35	35%
	Between 51 and 100	13	13%
	Between 101 and 500	12	12%
	More than 500	8	8%
Projects average duration	Between 1 and 3 months	14	14%
	Between 4 and 6 months	16	16%
	Between 7 and 12 months	34	34%
	Between 13 and 24 months	23	23%
	More than 24 months	14	14%
Quantity of project team	Less than 11 members	59	58%
	Between 11 and 15 members	16	16%
	Between 16 and 20 members	11	11%
	Between 21 and 25 members	1	1%
	Between 26 and 30 members	2	2%
	More than 30 members	12	12%
^1^ N = Number of respondents.			
